# Phylogeography of *Begonia luzhaiensis* suggests both natural and anthropogenic causes for the marked population genetic structure

**DOI:** 10.1186/s40529-019-0267-9

**Published:** 2019-09-06

**Authors:** Yu-Hsin Tseng, Han-Yau Huang, Wei-Bin Xu, Hsun-An Yang, Ching-I Peng, Yan Liu, Kuo-Fang Chung

**Affiliations:** 1 Research Museum and Herbarium (HAST), Biodiversity Research Center, Academia Sinica, Taipei, Taiwan; 2 School of Forestry and Resource Conservation, National Taiwan University, Taipei, Taiwan; 3 Guangxi Key Laboratory of Plant Conservation and Restoration Ecology in Karst Terrain, Guangxi Institute of Botany, Guangxi Zhuang Autonomous Region and Chinese Academy of Sciences, Guilin, Guangxi China

**Keywords:** *Begonia* sect. *Coelocentrum*, Chloroplast marker, EST-SSR, Flora of Guangxi, Glacial periods, Limestone karst conservation, Sino-Vietnamese limestone karsts (SVLK)

## Abstract

**Background:**

Sino-Vietnamese limestone karsts (SVLK) are a biodiversity hotspot rich in endemic plant species associated with caves and cave-like microhabitats. Based on phylogenetic studies of *Begonia* sect. *Coelocentrum*, a species-rich and characteristic SVLK clade, geographic isolation caused by extensive and continuous karstification was proposed as the major driving force triggering population diversification and geographic speciation. To test this proposition, population genetics and phylogeography of *Begonia luzhaiensis* were investigated using EST-SSR markers and the chloroplast *trnC*-*ycf6* intergenic spacer.

**Results:**

*F* statistics, Bayesian clustering analysis, AMOVA, and PCoA of both data sets all indicated substantial population differentiation and significant isolation by distance. Nested clade phylogeographic analyses inferred that historical fragmentations have been prominent, congruent with Guangxi’s geohistory of karstification as well as suggesting a mountain chain in northeastern Guangxi could have also acted as a major geographic barrier. A Bayesian skyline plot (BSP) indicated a slight decline in effective population size at 75,000 years ago (75 Kya), coinciding with the last glacial period during which the increased aridity in East Asia had retarded karstification, negatively affecting the populations of *B. luzhaiensis*. However, BSP detected a continuous and further population decline until the present time even though summer monsoons have resumed since the end of the last glacial maximum.

**Conclusions:**

The microevolution patterns of *B. luzhaiensis* support that limited gene flow would have greatly enhanced the effects of random genetic drift and has been a major factor promoting diversification in *Begonia*, highly congruent with previous proposition. Based our study, we further propose that the arrival of Paleolithic *Homo sapiens* whose activities centered around limestone caves could have had further impacts on the populations of *B. luzhaiensis*, resulting in additional population decline. Further habitat destruction could have resulted from the transition from hunter gathering to food-producing societies ca. 20–10 Kya and the development of agriculture ca. 10 Kya in South China. Implications of the current study for SVLK plant conservation are also discussed.

**Electronic supplementary material:**

The online version of this article (10.1186/s40529-019-0267-9) contains supplementary material, which is available to authorized users.

## Background

**Fig. 1:**
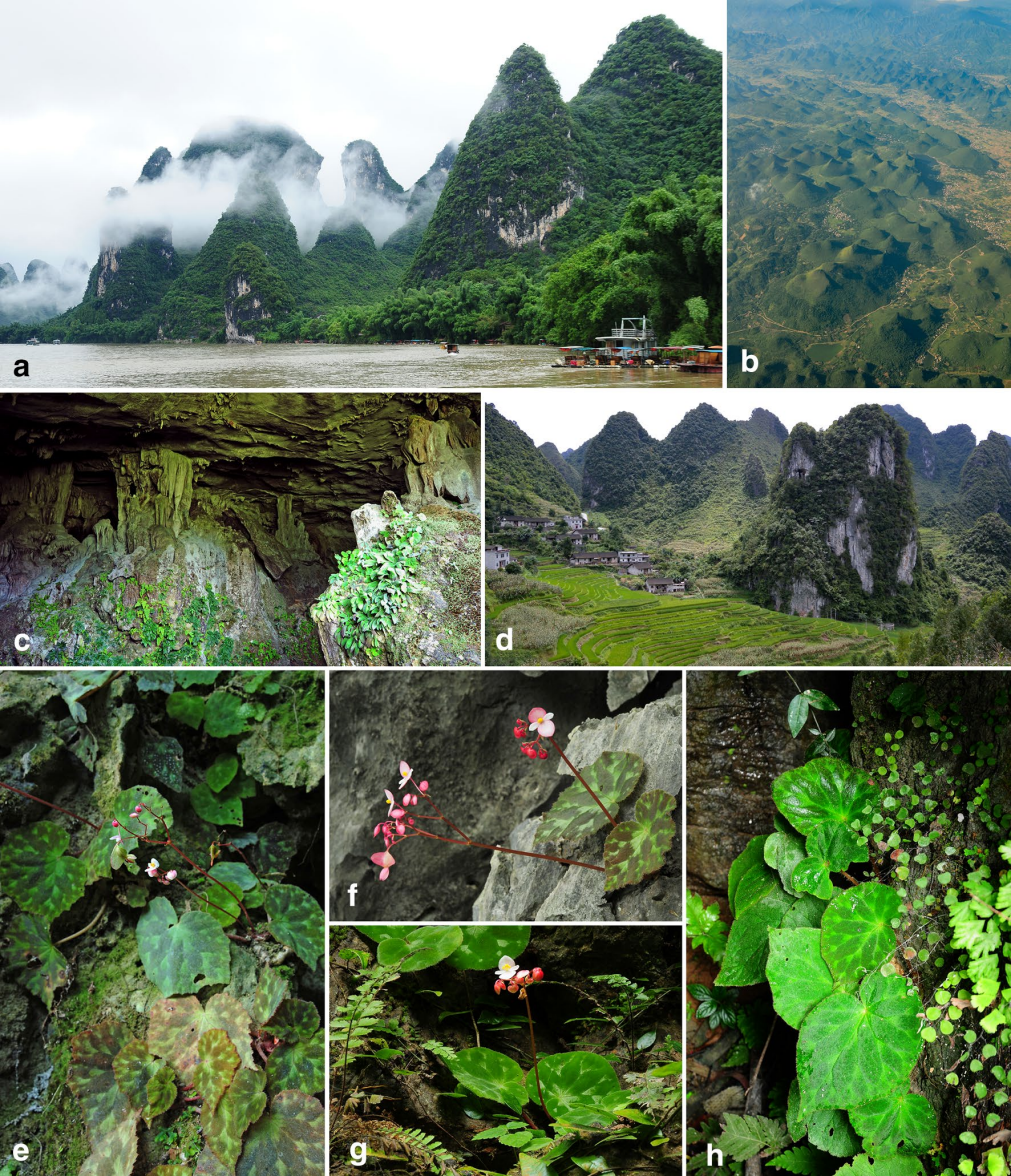
Guangxi’s limestone karsts (**a**–**d**) and habitats of *Begonia luzhaiensis* (**e**–**h**). **a** The picturesque limestone fengcong karst (Waltham 2008) along the Li River in Guangxi, China is a habitat for many limestone plants, including *B. luzhaiensis*. **b** Aerial photography taken before descending into Guilin Airport, Guangxi, showing the fragmented and cockpit-like karst landscape. **c** The entrance of a limestone cave in Luzhai County, Guangxi where the LZ population (Fig. 2) was sampled. This cave is also the type locality of one rare gesneriad, *Chirita luzhaiensis* (≡ *Primulina luzhaiensis*) (Huang et al. 2010). **d** Rice terraces in Tian’e County, Guangxi, showing the typical agricultural practices in the region and degraded limestone forest in a limestone karst. **e** *B. luzhaiensis* grows in the limestone cave (Fig. 1c) in Luzhai County (LZ in Fig. 2). **f** *B. luzhaiensis* grows on a limestone outcrop in Xincheng (XC in Fig. 2). **g** *B. luzhaiensis* grows in the entrance of a limestone cave in Rongshui County (RS in Fig. 2). **h** *B. luzhaiensis* grows on the base of a limestone tower along the Li River (YS in Fig. 2)

Karstification is the geomorphological process whereby soluble and porous bedrock (i.e., limestone, dolomite, gypsum, etc.) is dissolved and infiltrated by water via chemical and mechanical forces through which the surface features and subterranean drainage network of karsts are developed (Day and Urich 2000; Ford and Williams 2007; Williams 2008). In Southeast Asia and South China, more than 800,000 km^2^ of land are limestone karst, characterized by splendid towers [i.e., fengcong/cockpit karst (Waltham 2008)], peaks [i.e., fenglin/tower karst (Waltham 2008)], gorges, depressions, caves, underground tunnels, and subterranean rivers (Fig. 1a–d). Many of these scenic landscapes are not only UNESCO World Heritage Sites attracting tourists from around the world (Fig. 1a) (Day and Urich 2000; Williams 2008), but also major biodiversity hotspots rich in marvelous flora and fauna (Clements et al. 2006; Peng et al. 2014; Xu et al. 2017).

Stretching from South China to northern Vietnam, Sino-Vietnamese limestone karsts (SVLK) are particularly noted for the picturesque landscapes (Fig. 1a) and rich biodiversity with extremely high levels of endemicity. In China, 61 of the ca. 250 Chinese endemic plant genera are found only in Guangxi (Qin and Liu 2010), a province known for its spectacular limestone karsts (Sweeting 1978) (Fig. 1a), with a majority of these endemic genera associated with limestone ecosystems (Hou et al. 2010). The limestone flora of Guangxi is especially noted for a suite of plant genera with exceedingly highly levels of endemism and restricted distribution almost exclusively confined to the ‘twilight zone’ (Poulson and White 1969) of caves and cave-like microhabitats (Fig. 1c) (Xu et al. 2012; Chung et al. 2014; Monro et al. 2018). These narrowly distributed limestone plants—e.g., *Aspidistra* Ker Gawl., *Begonia* L., the fern genus *Polysticum* Roth, *Impatiens* L., and gesneriad genus *Primulina* Hance, etc.—also present intriguing study systems for investigating plant speciation (Chung et al. 2014). However, the major driving forces that created this plant diversity and the great richness of these narrow-endemic plants in caves of limestone karsts in South China remain poorly understood.

*Begonia* L. (Begoniaceae), comprising more than 1900 species (Moonlight et al. 2018), is the 5th or 6th largest flowering plant genus distributed in tropical and subtropical Asia, Africa and America. Of the 70 sections of *Begonia* (Moonlight et al. 2018), *Begonia* sect. *Coelocentrum* Irmsch. is a remarkable example of a limestone plant group with high endemism and species diversity (Gu et al. 2007; Chung et al. 2014; Qin et al. 2017b). Plants of this section (i.e., sect. *Coelocentrum* s.str.), characterized by their parietal placentation, are found in caves and cave-like microhabitats of SVLK (Fig. 1c), with the highest diversity in Guangxi (Gu et al. 2007; Chung et al. 2014). In the past two decades, explorations and taxonomic studies in the region have greatly expanded the number of species in the section from 15 (Ku 1999) to 78 (Chung et al. 2014; Peng et al. 2014, 2015a, b; Li et al. 2016; Qin et al. 2017b; Chen et al. 2018). As a great portion of SVLK remains unexplored, it is fully expected that further exploration will undoubtedly uncover more novelties (Chen et al. 2018).

Based on phylogenetic analyses of Asian *Begonia*, Chung et al. (2014) showed that all sampled species of rhizomatous SVLK *Begonia* are grouped in a strongly supported and yet internally poorly-resolved clade (Clade SVLB) dominated by sect. *Coelocentrum*, indicating its single evolutionary origin, radiation, and adaptation to limestone substrate and suggesting a strong phylogenetic niche conservatism (Wiens 2004) of Clade SVLB. Divergent times estimated by a Yule model in BEAST (Drummond et al. 2012) indicated that the mean divergent crown age of Clade SVLB was estimated to be 8.06 Mya (million years ago), with HPD (highest posterior density) date ranging from 5.09 to 11.21 Mya (Chung et al. 2014), corresponding to the onset of the East Asian monsoon triggered by the coeval uplift of the Himalaya-Tibetan Plateau (An et al. 2001). Geological studies suggest that, since the Miocene, the increased summer precipitation brought by the East Asian monsoon has provided the excessive moisture essential for karstification (Liu 1997; Liu et al. 2001). This geomorphological process that has generated the splendid Sino-Vietnamese limestone karsts would have also created copious cave and cave-like microhabitats suitable for the spread of limestone karst plants such as *Begonia* (Chung et al. 2014). As karstification proceeds, caves develop, collapse, and eventually diminish (Palmer 1991; Ford and Williams 2007), resulting in geographic isolation of limestone karst plants because strong niche conservatism would have forced limestone plants to track their ancestral niche (Wiens 2004; Kozak et al. 2006; Chung et al. 2014). These cave and cave-like microhabitats thus have functioned (Chung et al. 2014) as “*paleorefugia, now*-*fragmented relicts of a formerly widespread matrix community*” (Nekola 1999). Moreover, because *Begonia* species are generally poor dispersers (Hughes and Hollingsworth 2008) of low vagility (Kozak et al. 2006; Fouquet et al. 2007) prone to diversify via geographic isolation (Hughes and Hollingsworth 2008), restricted gene flow among conspecific populations could effectively lead to population differentiation (Slatkin 1993). Consequently, geographic fragmentation resulting from continuous karstification and restricted gene flow jointly would promote population differentiation in *Begonia* as well as other limestone karst plants (Gao et al. 2015), resulting in the proliferation of new species manifesting as species radiation in SVLK (Chung et al. 2014). However, because the major driving force of this evolutionary process is geographic isolation, this diversification pattern is better characterized as ‘non-adaptive radiation’ (Gittenberger 1991; Kozak et al. 2006; Rundell and Price 2009), a pattern reported in organisms of low vagility and strong niche conservatism (Gittenberger 1991; Comes et al. 2008).

**Fig. 2:**
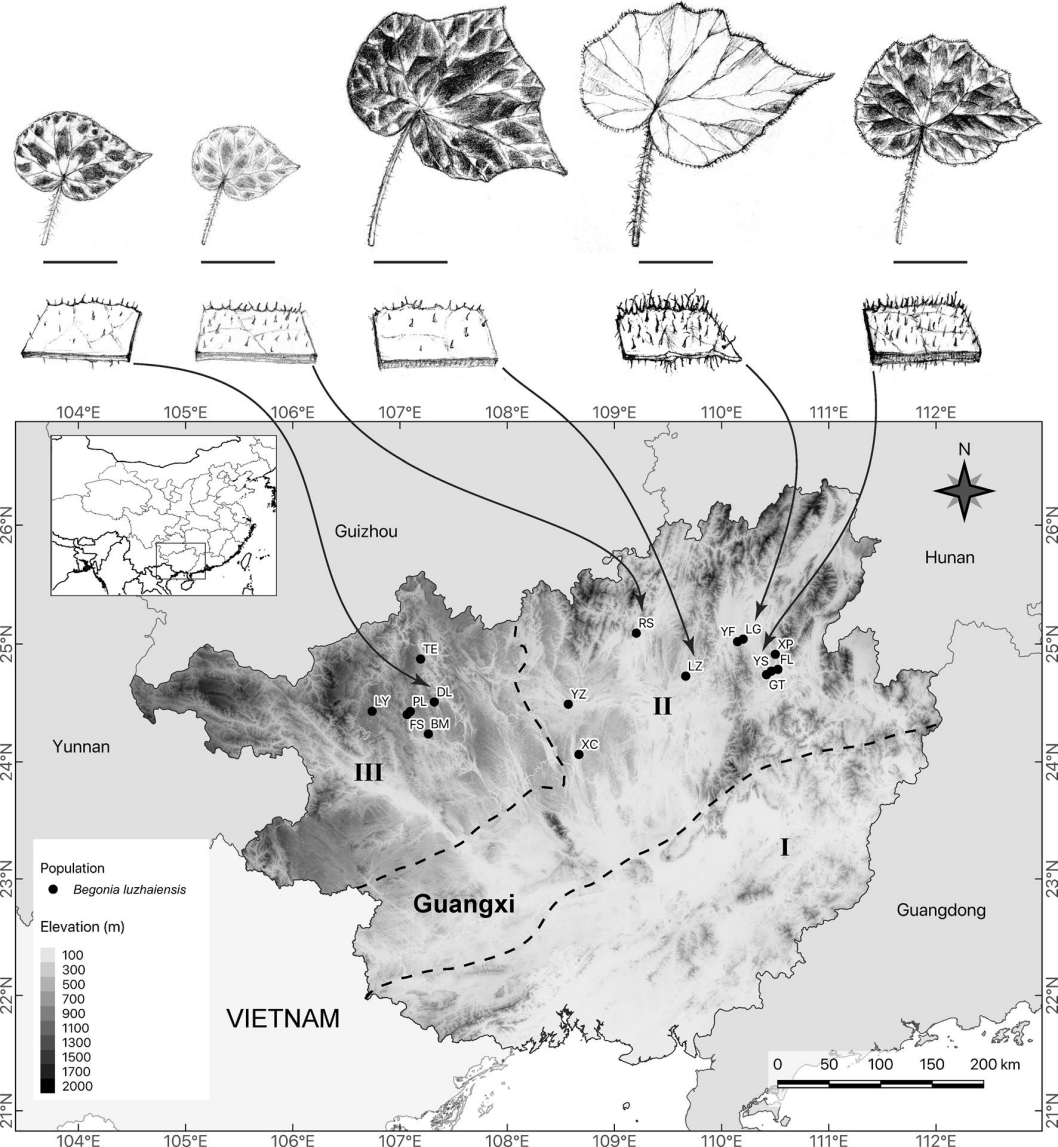
Sampling sites and leaf variation of *Begonia luzhaiensis*. Dash lines demarcate Liu et al. (2001)’s classification of Guangxi’s karst landforms: (I) karst plain, (II) Fenglin plain, and (III) Fengcong depression. Scale bar = 5 cm

The microevolutionary scenario postulated by Chung et al. (2014) predicts a marked population genetic structure and phylogeographic pattern in SVLK plant species inhabiting caves and cave-like microhabitats. The scenario predicted by Chung et al. (2014) has been supported by analyses of the *Primulina eburnea* (Hance) Yin Z.Wang species complex that is distributed in limestone karsts of southern China (Gao et al. 2015). To further test these hypotheses, expressed sequence tag-simple sequence repeat (EST-SSR) markers (Tseng et al. 2017) and cpDNA sequences of *trn*C-*ycf*6 intergenic spacer were collected for *Begonia luzhaiensis* T.C.Ku (Fig. 1e–h). *Begonia luzhaiensis* is the only species of sect. *Coelocentrum* s.str. with a relatively wide distribution (Fig. 2) suitable for phylogeographic and population genetic analyses; the rest species of Clade SVLB are known from a single or a few localities except for *B. cavaleriei* H.Lév. and *B. leprosa* Hance that were formerly classified under sect. *Diploclinium* and sect. *Leprosae*, respectively (Ku 1999; Gu et al. 2007). As limestone ecosystems are increasingly threatened worldwide, especially in Southeast Asia (Clements et al. 2006; Hughes 2017) and South China (Monro et al. 2018), the extent of this species’ genetic diversity inferred from the present study will also provide important insights into conservation and management strategies for the precious Sino-Vietnamese limestone karst flora.

## Methods

### Sample collection

*Begonia luzhaiensis* is not included in the “Threatened Species List of China’s Higher Plants” (Qin et al. 2017a). For the purpose of scientific research, permits were not required by authorities of the collecting areas. *Begonia luzhaiensis* is endemic to Guangxi (Gu et al. 2007). Consulting Liu et al. (2001)’s classification of the regional tropical karst landform of Guangxi, the distribution range of *B. luzhaiensis* falls within (II) the Fenglin plain and (III) the Fengcong depression, absent from (I) the karst plain (Fig. 2). *Begonia luzhaiensis* is easily recognizable by its distinct variegation and maculation on leaves (Cui and Guan 2013); however, considerable variation exists across its distribution range (Figs. 1e–h and 2). For example, compared with other populations of *B. luzhaiensis*, plants from the Luzhai (LZ) and Yangshuo (YS) usually have large and hairy leaves, while leaves of plants from Donglan (DL) are darker and less hairy (Fig. 2). Although *B. luzhaiensis* is the only species in *Begonia* sect. *Coelocentrum* s.str. that has a relatively wide distribution range, its population sizes vary greatly among localities, ranging from a few individuals to several hundred plants per site. Based on herbarium records at HAST, IBK, and PE, a total of 165 individuals of *B. luzhaiensis* were collected from 15 localities (Table 1; Fig. 2), covering almost all known populations of the species. One plant collected from Yizhou (YZ) in 2002 and cultivated in the greenhouse of Guangxi Institute of Botany was also included (Fig. 2). Our attempt to sample the Yizhou population failed due to habitat destruction. Fresh leaves were collected and preserved in silica-gel. Voucher specimens were deposited at the Herbarium (HAST) of Biodiversity Research Museum, Academia Sinica, Taiwan (Table 1). *Begonia asteropyrifolia* Y.M.Shui & W.H.Chen, a species closely related to *B. luzhaiensis*, was chosen as an outgroup.

**Table 1:** Voucher information for samples used and GenBank accession numbers of cpDNA sequences

Population (Code)	Voucher (HAST specimen ID)	Collection locality	Geographic coordinates	GenBank accession number
*Begonia luzhaiensis* T.C.Ku				
Bama (BM)	*S.*-*M. Ku 2022* (HAST 142361)	Bana Village, Xishan Township, Bama County, Guangxi, China	24°14′17″N 107°15′50″E	MG063376
Donglan (DL)	*S.*-*M. Ku 2002* (HAST 142357)	Dawei Village, Donglan Town, Donglan County, Guangxi, China	24°30′33.0″N 107°19′13.0″E	MG063379, MG063380
Fengshan (FS)	*S.*-*M. Ku 2016* (HAST 142358)	Paoli Township, Fengshan County, Guangxi, China	24°24′14.0″N 107°04′00.0″E	MG063378
Fuli (FL)	*H.*-*Y. Huang* et al*. 6* (HAST 142351)	Fuli Town, Yangshuo County, China	24°47′08.2″N, 110°31′22.5″E	MG063293–MG063297
Gaotian (GT)	*K.*-*F. Chung* et al*. 1855* (HAST 142364)	Paitou Village, Gaotian Town, Yanshuo County, Guangxi, China	24°44′25.8″N 110°25′04.9″E	MG063369–MG063373
Lingui (LG)	*H.*-*Y. Huang* et al*. 31* (HAST 142353)	Sishan Village, Huixian Town, Lingui County, Guangxi, China	25°02′27.9″N 110°11′59.4″E	MG063323–MG063327
Lingyun (LY)	*C.*-*I Peng* et al*. 24344* (HAST 138438)	Luolou Town, Lingyun County, Guangxi, China	24°25′48″N, 106°44′24″E	MG063381–MG063415
Luzhai (LZ)	*H.*-*Y. Huang* et al*. 18* (HAST 142354)	Zhongdu Town, Luzhai County, Guangxi, China	24°43′37.9″N 109°39′50.6″E	MG063311–MG063322
Paoli (PL)	*S.*-*M. Ku 2018* (HAST 142359) & *2019* (HAST 142360)	Paoli Township, Fengshan County, Guangxi, China	24°25′43.0″N 107°05′46.0″E	MG063374, MG063375
Rongshui (RS)	*H.*-*Y. Huang* et al*. 33* (HAST 142355)	Xidong Scenic Area, Ronshui County, Guangxi, China	25°05′30.2″N 109°12′10.7″E	MG063334–MG063347
Tian’e (TE)	*K.*-*F. Chung* et al*. 1825* (HAST 142363)	Dulou Village, Bamu Township, Tian’e County, Guangxi, China	24°52′21.2″N 107°11′31.0″E	MG063366–MG063368
Xincheng (XC)	*H.*-*Y. Haung* et al*. 102* (HAST 142356)	Chengguan Town, Xincheng County, Guangxi, China	24°03′49.5″N 108°40′08.9″E	MG063348–MG063365
Xingping (XP)	*K.*-*F. Chung* et al*. 3017* (HAST 142365)	Xingping Town, Yangshou County, Guangxi, China	24°54′48.3″N 110°30′02.9″E	MG063416–MG063424
Yongfu (YF)	*C.*-*I Peng* et al*. 24374* (HAST 138467)	Luojin Township, Yongfu County, Guangxi, China	25°01′12.0″N 110°09′02.0″E	MG063328–MG063333
Yangshuo (YS)	*H.*-*Y. Huang* et al*. 8* (HAST 142352)	Yanshuo Town, Yanshuo County, Guangxi, China	24°46′12.9″N, 110°27′43.4″E	MG063298–MG063310
Yizhou (YZ)	*Y.*-*M. Shui 9010* (HAST 94959)	Yankou Village, Yizhou District, Guangxi, China	24°29′24.0″N, 108°34′12.0″E	MG063377
*Begonia asteropyrifolia* Y.M.Shiu & W.H.Chen				
(Outgroup)	*C.*-*I Peng* et al*. 21062* (HAST 117107)	Donglan Town, Donglan County, Guangxi, China	–	MG063292

All voucher specimens are deposited in HAST. The HAST specimen ID is encrypted with the hyperlink to its digital image

### Microsatellite genotyping and DNA sequencing

To overcome difficulty of extracting quality DNA of *Begonia* species, a modified CTAB protocol optimized by Kopperud and Einset (1995) was followed (e.g., Chung et al. 2014). Samples from 15 populations were genotyped by 16 EST-SSR markers using primer pairs developed for *B. luzhaiensis* (Tseng et al. 2017). Amplification and genotyping conditions followed the protocol described in Tseng et al. (2017). Based on our preliminary tests for marker variability as well as efficiency of PCR amplification, the *trn*C-*ycf*6 intergenic spacer of the chloroplast genome was chosen for phylogeographic analysis. Total genomic DNA was extracted from the silica gel-dried leaves using the protocols outlined in Chung et al. (2014). The PCR reaction was performed in 20 μL of final volumes with10 µL of 2 × Master Mix Red (Ampliqon, Odense, Demark), 1 μL of each specific primer (10 μM each), and 30 ng template. Primers for the *trn*C-*ycf*6 region amplification were “trnC” (5′- CCAGTTCAAATCTGGGTGTC-3′) (Demesure et al. 1995) and “petN1r” (5′-CCCAAGCAAGACTTACTATATCC-3′) (Lee and Wen 2004). The PCR amplifications were initiated with set at: 94 °C for 5 min, 32 cycles of 94 °C for 40 s, 53 °C for 35 s and 72 °C for 1 min and a final extension in 72 °C for 5 min. PCR products were purified using QIAquick PCR purification Kit (Qiagen, Valencia, California, U.S.A.) and sequenced using an ABI PRISM dye terminator cycle sequencer, model 3700 (Applied Biosystems, Foster City, California, U.S.A.). All sequences were deposited in GenBank with accession numbers MG063292–MG063424.

### Analysis of EST-SSR

The allelic richness (*A*_r_), gene diversity (*H*_s_), observed heterozygosity (*H*_O_), expected heterozygosity (*H*_E_), and inbreeding coefficient (*F*_is_) of EST-SSR markers were computed using FSTAT 2.9.3 (Goudet 2001). Tests for deviation from Hardy-Weinberg equilibrium (HWE) and linkage disequilibrium (LD) for each EST-SSR loci were estimated by GENEPOP 4.2 (Rousset 2008). For each locus, the FDIST method (Beaumont and Nichols 1996) implemented in LOSITAN (Antao et al. 2008) was used to test for selection. Haplotype-based genetic differentiation (*F*_st_) between populations was calculated using permutations in ARLEQUIN 3.5.1.2 (Excoffier and Lischer 2010).

The population structure was evaluated by the software STRUCTURE 2.3.4 (https://web.stanford.edu/group/pritchardlab/structure.html) (Pritchard et al. 2000) and principal coordinate analysis (PCoA) using GENALEX 6.5 (Peakall and Smouse 2012). In STRUCTURE, the possible number of clusters (*K*) was set to vary from 1 to 15 with 10 independent runs. The analysis was performed assuming the admixture model and correlated allele frequencies among clusters. Each run consisted of 1,000,000 steps Markov chain Monte Carlo (MCMC) replicates after a 100,000 steps burn-in (Evanno et al. 2005). The best grouping was evaluated by Δ*K* (Evanno et al. 2005) in STRUCTURE HARVEESTER v 0.6.94 (Earl and Vonholdt 2012). Alignment of cluster assignments across replicate analyses was conducted in CLUMPP (Jakobsson and Rosenberg 2007). In PCoA, *Nei*’s pairwise genetic distances were calculated and visualized by PCoA using GENALEX (Peakall and Smouse 2012).

**Fig. 3:**
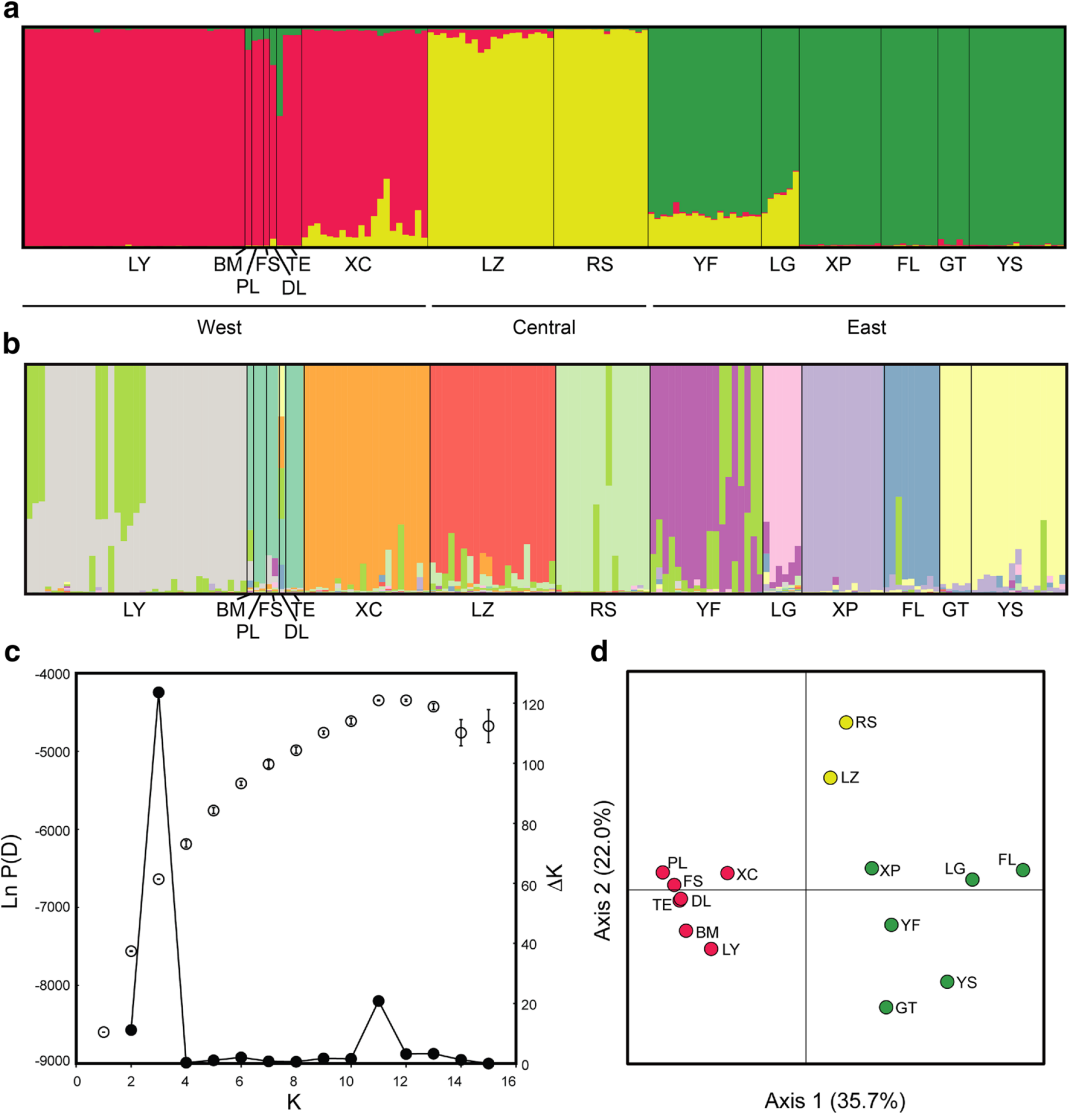
The results of STRUCTURE & PCoA. **a** STRCTURE bar plot representing K = 3 of the Bayesian assignment of 165 individuals from 15 populations of *Begonia luzhaiensis* based on genetic variation of 16 EST-SSR loci. Each vertical bar represents an individual plant is represented by a single vertical line grouped by populations. **b** STRCTURE bar plot representing K = 11. **c** The *ΔK* plot shows that *K* = 3 is the highest *ΔK* value. **d** PCoA analysis is based on EST-SSR loci using GENALEX

To calculate genetic variation, populations of *B. luzhaiensis* were grouped into East (FL, GT, LG, XP, YF and YS), Central (LZ and RS), and West (BM, DL, FS, LY, PL, TE, XC and YZ) regions based on the grouping results of STRUCTURE and PCoA (Fig. 3). The analysis of molecular variance (AMOVA) was used to test genetic variation between areas (groupings based on the results of Structure and PCoA) and among and within populations. A non-grouped analysis was also conducted. The AMOVA was also assessed by ARLEQUIN using *R*-statistics with significance tests by 10,000 non-parametric random permutations (Excoffier and Lischer 2010).

To test for the assumption of isolation by distance (IBD), a Mantel test was performed using genetic and geographic distances. Pairwise *F*_st_ between populations used as the genetic distance metric was calculated based on *Nei*’s approach (Nei et al. 1977) using GENALEX. Geographic distances between location pairs were calculated using Geographic Distance Matrix Generator v1.2.3 (Ersts 2011). Mantel tests and Mantel correlogram test were carried out by regressing *F*_st_/(1 − *F*_st_) against the natural logarithm of geographical distance using the vegan package in R (Oksanen et al. 2010) with 9999 permutations.

### Analysis of *trn*C-*ycf*6 spacer data

Raw sequences of *trn*C-*ycf*6 spacer were edited and assembled by SeqMan™ II (DNASTAR, USA) with subsequent manual adjustments. DNA sequences were aligned by MUSCLE v3.6 (Edgar 2004) implemented in MEGA 5 (Tamura et al. 2011) using a first round of multiple alignment and posterior rounds of refinement under default setting. Alignments were subjected to manual adjustments in Mesquite v.3.0.3 (Maddison and Maddison 2018). Indels were treated as the fifth character when calculating the indices of genetic diversity. The program DnaSP v5.0 (Librado and Rozas 2009) was used to calculate the degree of polymorphism (π), haplotype diversity (*h*), and *θ* estimated by segregating sites (S) for populations with at least three samples (Table 2).

**Table 2:** Genetic diversity and demographic characteristic of *Begonia luzhaiensis* based on EST-SSR markers and *trnC*-*ycf6* spacer sequence

Population code	*n* (SSR/cpDNA)	SSR					cpDNA			
		*A*_r_	*H*_s_	*F*_is_	*H*_o_	*H*_e_	*π*	*h*	θw	Haplotype (*n*)
East Guangxi										
Fuli (FL)	9/5	1.552	0.553	0.039	0.531	0.518	0.000	0.000	0.000	H2 (5)
Gaotian (GT)	5/5	1.478	0.472	-0.106	0.522	0.429	0.000	0.000	0.000	H4 (5)
Lingui (LG)	6/5	1.371	0.383	0.228	0.296	0.336	0.171	0.600	0.137	H3 (2), H5 (3)
Xingping (XP)	13/9	1.266	0.266	0.080	0.245	0.255	0.000	0.000	0.000	H1 (9)
Yongfu (YF)	18/6	1.261	0.264	0.212	0.208	0.251	0.000	0.000	0.000	H3 (6)
Yangshuo (YS)	15/13	1.475	0.476	0.073	0.441	0.459	0.000	0.000	0.000	H2 (13)
Central Guangxi										
Luzhai (LZ)	20/12	1.482	0.487	0.187	0.396	0.455	0.000	0.000	0.000	H6 (12)
Rongshui (RS)	15/14	1.326	0.328	0.142	0.282	0.313	0.000	0.000	0.000	H7 (14)
West Guangxi										
Bama (BM)	1/1	–	–	–	–	–	0.000	0.000	0.000	H8 (1)
Donglan (DL)	1/2	–	–	–	–	–	0.142	1.000	0.142	H6 (1), H9 (1)
Fengshan (FS)	1/1	–	–	–	–	–	0.000	0.000	0.000	H9 (1)
Lingyun (LY)	35/35	1.236	0.237	0.201	0.189	0.232	0.000	0.000	0.000	H8 (35)
Paoli (PL)	2/2	1.073	0.067	-0.500	0.094	0.055	0.000	0.000	0.000	H9 (2)
Tian’e (TE)	4/3	1.245	0.266	0.569	0.115	0.212	0.000	0.000	0.000	H8 (3)
Xincheng (XC)	20/18	1.294	0.295	0.101	0.265	0.285	0.000	0.000	0.000	H6 (18)
Yizhou (YZ)	-/1	–	–	–	–	–	0.000	0.000	0.000	H6 (1)

*n* (SSR/cpDNA), number of individuals sampled for SSR/cpDNA; A_R_: mean allelic richness corrected for sample size; H_S_: gene diversity; F_IS_: inbreeding coefficient; H_O_: observed heterozygosity; H_E_: expected heterozygosity; π: nucleotide diversity; h: haplotype diversity

Haplotype-based genetic differentiation (*F*_st_) between populations was calculated and its significance was tested using permutations in ARLEQUIN (Excoffier and Lischer 2010). To determine whether the genetic distance between populations was determined by geographic distance, isolation by distance analysis was performed using GENALEX (Peakall and Smouse 2012) as described above for EST-SSR. The analysis of molecular variance (AMOVA) was performed by ARLEQUIN to estimate the partitioning of genetic variation among areas (groupings based on STRUCTURE and PCoA’s results) within populations and among populations.

To estimate the dynamics of population size fluctuations over time, the Bayesian Skyline Plot (BSP) method, a coalescent-based approach utilized standard MCMC integration to evaluate the posterior probability distribution of effective population size, was performed to estimate the historical demographic change of *B. luzhaiensis* using BEAST v1.8.4 (Drummond et al. 2012). Some recent studies (Chikhi et al. 2010; Heller et al. 2013) showed that population structure tends to produce a false signal of population bottleneck with some sampling schemes in demographic history inferences, but different sampling strategies can help to overcome this structure effect (Chikhi et al. 2010; Heller et al. 2013; Mazet et al. 2015, 2016). Therefore, we used four sampling strategies based on Heller et al. (2013) and Sgarlata et al. (2018) to perform the historical demographic change in BSP, including (1) local sampling: all samples from a spatial cluster (eastern, central, western regions based on the grouping results of PCoA and STRUCTURE), (2) pooled sampling: three to five randomly picked samples from each population, excluding populations with only one or two individuals, (3) scattered sampling: one randomly picked sample from each population, and (4) structured sampling: all samples. For each sampling strategy, the substitution rate of *trn*C-*ycf*6 spacer was set as 1.01 × 10^9^ (per site per year) referring to previous reports of cpDNA for herbaceous plants with a normal distribution (Graur and Li 2000). Based on Akaike information criteria (AIC), the J-Model test (Posada 2008), calculated through the CIPRES web-server (http://www.phylo.org/index.php/portal/), selected HKY as the most appropriate substitution model for *trn*C-*ycf*6. Prior distribution for Bayesian skyline, population parameter sizes were uniform, with an initial value of 1 × 10^5^, a lower value of 100 and upper value of 2 × 10^6^. Two independent MCMC runs were conducted in the analyses, each with 100 million generations and the trees were sampled every 1000 generations, with first 25% discard as burn-in. Time series plots of all parameters were analyzed in Tracer v.1.5 (Rambaut et al. 2018) to check for effective sample size values that were larger than 200.

Demographic history was explored using two different methods. First, Tajima’s *D* (Tajima 1989) and Fu’s *Fs* (Fu 1997) were calculated using ARLEQUIN to infer demography by testing deviations from the neutral equilibrium condition. Second, the mismatch distribution was implemented to identify the population expansion model using ARLEQUIN. We estimated demographic expansion factor (*τ*), mutation parameter (*θ*), and number of migrants (*M*) at 95% confidence intervals. The sum of squared deviations (*SSD*) was estimated to test the goodness-of-fit of the observed and expected mismatch distributions (Schneider and Excoffier 1999) and significance was assessed after 10,000 permutations. The expansion time (*T*) was computed with the equation *T* = *τ/*2*u*, where *u* is the mutation rate for the entire sequence. The value of *u* is derived from *u* = *µm*, where the *µ* is the mutation rate per site per generation, and *m* is the number of investigated nucleotides. The generation time of *B. luzhaiensis* was assumed to be 2 years based on our observation in the experimental greenhouse of the Academia Sinica.

To test the null hypothesis of no association between geography and haplotype variation, nested clade phylogeographic analysis (NCPA) was performed (Templeton et al. 1995, 2009; Buhay et al. 2009; Santos et al. 2017; Bekker et al. 2018), with cautions taken from criticisms against NCPA (e.g., Knowles 2008). Haplotype network was reconstructed using TCS v.1.21 with the 95% parsimony criterion (Clement et al. 2000) using *B. asteropyrifolia* as an outgroup. A series of multi-level nested clade designs was defined following the rules of Templeton and Sing (1993) based on the network constructed by TCS. Subsequently, the program GeoDis (Posada et al. 2000) was applied to calculate the clade distance (*D*_*c*_), the nested clade distance (*D*_*n*_), and interior-tip statistic (I-T) to test the null hypothesis of no geographic association of clades and nested clades, with 95% confidence level and 10,000 permutations. These measures were interpreted by referring to the latest inference key from GeoDis website (http://darwin.uvigo.es/software/geodis.html).

## Result

### Sample collection

A total of 165 plants from 15 populations were successfully genotyped using EST-SSR markers and 131 alleles were detected across the 16 loci (Table 2).

### Analyses of EST-SSR

The mean number of alleles per locus ranged from 1.1 (BM, FS, and PL) to 3.9 (YS) with a mean of 2.3 ± 0.9. Within populations, the allelic richness (*A*_r_) ranged from 1.073 (PL) to 1.552 (FL) and gene diversity (*H*_s_) from 0.067 (PL) to 0.553 (FL). Expected heterozygosity was highest in population FL (0.518) and lowest in population PL (0.055), with a mean of 0.317 ± 0.131 (Table 1). The global *F*_st_ based on EST-SSR loci was 0.527 (*P* < 0.001) (Additional file 1: Table S1) and pairwise *F*_st_ between populations were also high (0.315–0.676) (Additional file 1: Table S3).

Prior to estimating further population genetic parameters, neutrality tests for outlier analysis for EST-SSR was conducted by FDIST. Because the null hypothesis of neutrality was not rejected by the result of outlier analysis, the EST-SSR markers used in this study were used for further population genetic assessment (Additional file 1: Table S1).

The ΔK statistic (Evanno et al. 2005) of the STRUCTURE analyses had a peak at K = 3, with a second and much lower peak at K = 11 (Fig. 3c). At K = 3, STRUCTURE identified clusters corresponding well to East Guangxi (YS, GT, FL, XP, LG, and YF), Central Guangxi (RS and LZ) and West Guangxi (XC, TE, DL, FS, PL, BM, and LY), with some admixtures detected in LG, YF, and LZ, and XC (Fig. 3a). This geographic clustering also corresponded well to the PCoA plot (Fig. 3d), in which the first and second axes extracted 35.7% and 22.0% of the total genetic variation, respectively. At K = 11, STRUCTURE identified 10 clusters, breaking the East Guangxi into five clusters (YS + GT, FL, XP, LG, and YF), Central Guangxi into two clusters (RS and LZ), and the West Guangxi into three clusters (XC, TE + DL + FS + PL + BM, and LY) with most clusters showing various degrees of admixture (Fig. 3b).

### Analysis of *trn*C-*ycf*6 spacer data

For chloroplast *trn*C-*ycf*6 spacer, DNA sequences of 132 plants from 16 populations were collected. The length of sequences ranged from 567 to 572 bp, with the aligned length of 601 bp. The sequence length of the outgroup species *B. asteropyrifolia* was 567 bp. Among all the populations examined, nucleotide diversity ranged from 0 (FL, YS, GT, YF, XP, LZ, RS, FS, PL, BM, TE, XC, YZ, LY) to 0.002 (LG, DL) and haplotype diversity (*h*) from 0 (FL, YS, GT, YF, XP, LZ, RS, FS, PL, BM, TE, XC, YZ, and LY) to 0.6 (LG) (Table 2). Pairwise *F*_st_ values between populations were low (0–0.009, Additional file 1: Table S3).

**Fig. 4:**
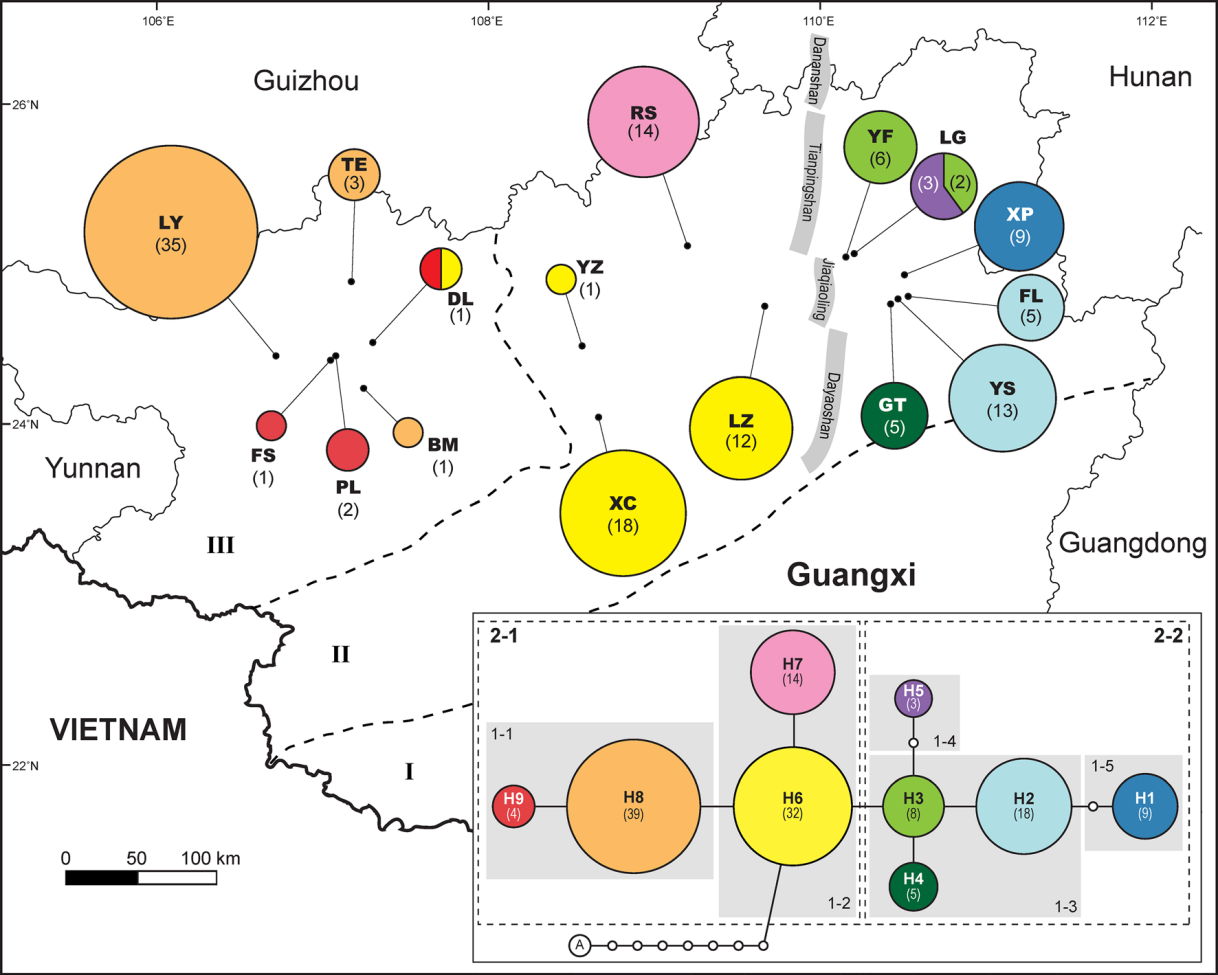
Distribution of *trnC*-*ycf6* haplotypes, haplotype network, and nested clade design in *B. luzhaiensis*. Statistical parsimony network was generated by TCS 1.21 rooted by *B. asteropyrifolia* (A). The size of circles is proportional to haplotype frequency, which is also indicated in the circles. Dash lines demarcate three types (I, II, and III in Fig. 2) of karst landforms in Guangxi based of Liu et al. (2001)’s classification

Nine haplotypes of *trn*C-*ycf*6 spacer (H1 to H9) in *B. luzhaiensis* were identified from 16 populations (Table 1; Fig. 4). Most populations have only one haplotype except for DL and LG that possess two shared haplotypes (DL: H6 and H9; LG: H3 and H5). Haplotype H1, H4 and H7 were distributed exclusively in population XP, GT and RS, respectively. In the statistical parsimony network, haplotypes were inter-connected by one or two mutations steps. Haplotype H6, identified as the ancestral haplotype for its highest outgroup probability (outgroup weight = 0.338) and connection with the outgroup *B. asteropyrifolia*, is distributed in central area (DL, LZ, XC, and YZ) of the distribution range of *B. luzhaiensis*, connected to the haplotypes of western (H8, H9) and eastern (H1 to H5) populations and the haplotype H7 in RS to the north (Fig. 4).

### Population structure and phylogeographic analyses

The hierarchical analysis of molecular variance (AMOVA) in non-grouping analysis revealed the higher variations among populations than within population both in EST-SSR data (56.28%, *P* < 0.001; 43.72%, *P* < 0.001) and cpDNA sequence (63.46%, *P* < 0.001; 36.54%, *P* < 0.001). However, in the grouping analysis, the highest proportion of molecular variance in the EST-SSR data was found among grouping (48.32%, *P* < 0.001), while the highest variance in cpDNA sequence was among population (51.89%, *P* < 0.001) (Table 3).

**Table 3:** Analysis of molecular variance (AMOVA) for *Begonia luzhaiensis* based on EST-SSR loci and *trnC*-*ycf6* spacer sequence (cpDNA)

Source of variation	EST-SSR					cpDNA				
	d.f.	Sum of squares	Variance components	Fixation indices	Percentage of variation	d.f.	Sum of squares	Variance components	Fixation indices	Percentage of variation
(a) Non-grouping										
Among populations	2	196.28	0.932		56.288*	2	65.36	0.782		63.46**
Within populations	321	232.25	0.724	*F*_*st*_ = 0.563	43.72**	128	57.66	0.450	*F*_*st*_ = 0.635	36.54**
Total	323	429.53	1.656			130	123.02	1.232		
(b) Grouping^a^										
Among groups	2	196.28	0.798	*F*_*ct*_ = 0.483	48.32**	2	65.36	0.563	*F*_*ct*_ = 0.457	44.73**
Among populations within groups	9	81.32	0.369	*F*_*sc*_ = 0.433	22.38**	13	54.26	0.640	*F*_*sc*_ = 0.956	51.89**
Within populations	312	150.94	0.484	*F*_*st*_ = 0.707	29.30*	116	3.40	0.029	*F*_*st*_ = 0.976	2.38**
Total	323	428.53	1.651			130	123.02	1.233		

F_st_: fixation index within population; F_sc_: fixation index among populations within groups; F_ct_: fixation index among groups

* *P *< 0.05, ** *P *< 0.001

^a^Grouping based on Structure and PCoA’s results

**Fig. 5:**
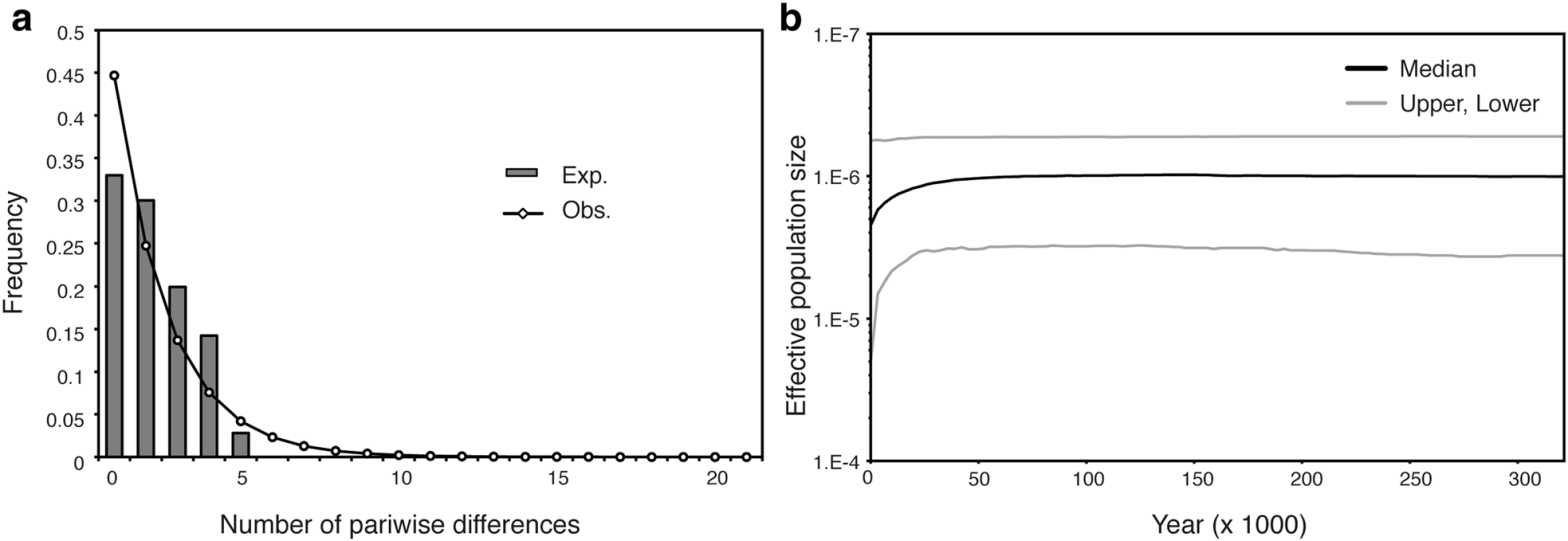
Historical demography of *B. luzhaiensis* inferred from *trnC*-*ycf6* sequence. **a** Pairwise mismatch distribution. **b** Bayesian skyline plot of structured sampling, showing the median and 95% highest posterior density interval

The value of Tajima’s D and Fu’s F were not significantly different from 0 in the total sample and for each region (Table 4). The mismatch distribution analysis revealed population expansion events in *B. luzhaiensis* based on the non-significant *SSD* (0.002, *P* = 0.758). Given the expansion parameter (*τ *= 1.161) and equation *T *= *τ*/2*u*, populations of *B. luzhaiensis* were estimated to expand 2.047 Mya (95% confidence interval = 0.596–6.072 Mya) (Table 5). A similar scenario was observed in the unimodal distribution of pairwise nucleotide differences (Fig. 5a), which also suggested that populations experienced demographic expansions (Additional file 2: Fig. S1).

**Table 4:** Tajima’s *D* and Fu’s *F*_*s*_ statistics for *Begonia luzhaiensis* based on *trnC*-*ycf6* spacer sequence

	East Guangxi	North Guangxi	West Guangxi	Total
Tajima’s *D* (*P* value)	0.403 (0.706)	1.591 (0.977)	− 0.540 (0.244)	0.485 (0.642)
Fu’s *F*_*s*_ (P-value)	1.167 (0.737)	1.619 (0.745)	0.728 (0.634)	1.172 (0.705)

**Table 5:** Estimated spatial expansion parameters of *τ*, *θ* and M for *Begonia luzhaiensis* based on *trnC*-*ycf6* spacer sequence

*τ*	*θ*	*M*	*SSD* (P-value)	Time of expansion (Ma)
1.161 (0.338–3.443)	0.361 (0.001–1.333)	6.625 (0.333–1047.893)	0.002 (0.758)	2.047 (0.596–6.072)

These parameters were obtained from the mismatch distribution under the spatial expansion model. Value in parentheses indicate 95% confidence intervals

SSD: Sum of squared deviation

For four of the six sampling strategies [structured (Fig. 5b), east in local (Additional file 3: Fig. S2A), pooled (Additional file 3: Fig. S2D), and scattered (Additional file 3: Fig. S2E)], the BSP results showed that populations of *B. luzhaiensis* experienced a long history of constant population size since the late Pleistocene until ca. 75 Kya when slight decrease was detected, with continuous and further decline until the present time (Fig. 5b and Additional file 3: Fig. S2D). For western and central regions in local sampling, the results showed a constant population size within the last 400 years (Additional file 3: Figs. S2B and C). Mantel test showed that there was an overall significant positive correlation between genetic (Additional file 1: Tables S3 and S4) and geographical distances (Additional file 1: Table S5) in both cpDNA and EST-SSR, and the signal was stronger for SSR (*Rxy* = 0.589, *P* < 0.001) than for cpDNA (*Rxy* = 0.38, *P* < 0.01) (Table 6).

**Table 6:** Summary for Mantel tests

	*R*_*xy*_	*P*
EST-SSR	0.589	0.001
cpDNA	0.38	0.001

The statistics of the nested clade phylogeographic analyses (NCPA) is summarized in Table 7. Based on NCPA, the null hypothesis of no geographic association of haplotype variation was rejected in all six clades with geographic and genetic variation (Fig. 4; Table 7). By applying the inference key, historical events appear to be the major mechanisms shaping the phylogeography of *Begonia luzhaiensis*, with allopatric fragmentation detected in Clades 1-2, 1-3, and the total cladogram, and past fragmentation and/or long distance colonization in Clades 2-1 and 2-2. In Clade 1-1, restricted gene flow with isolation by distance is inferred (Table 7).

**Table 7:** The result of nested clade phylogeographic analysis of *Begonia luzhaiensis* based on *trnC*-*ycf6* haplotypes

Clade	Significant clades and I-T	Significant parameter	Phylogeographic inferences
1-1	H8 (I) H9 (T) I-T	*D*_*c*_> ; *D*_*n*_> *D*_*c*_> ; *D*_*n*_>	1 → 2→3 → 4→No: Restricted gene flow with isolation by distance
1-2	H6 (I) H7 (T) I-T	*D*_*n*_> *D*_*c*_< ; *D*_*n*_< *D*_*c*_> ; *D*_*n*_>	1 → 19 → No: Allopatric fragmentation
1-3	H2 (I) H3 (I) H4 (T)	*D*_*c*_< ; *D*_*n*_< *D*_*c*_< ; *D*_*n*_> *D*_*c*_<	1 → 19 → No: Allopatric fragmentation
2-1	1-1 (T) 1-2 (I) I-T	*D*_*c*_< ; *D*_*n*_< *D*_*c*_< ; *D*_*n*_> *D*_*c*_> ; *D*_*n*_>	1 → 2→3 → 5→15 → No → 21 → No: past gradual range expansion followed by fragmentation or a past larger range followed by extinction in intermediate areas
2-2	1-3 (I) 1-4 (T) 1-5 (T) I-T	*D*_*n*_> *D*_*c*_< ; *D*_*n*_< *D*_*c*_>	1 → 2→3 → 5→15 → No → 21 → No: past gradual range expansion followed by fragmentation or a past larger range followed by extinction in intermediate areas
Total	2-1 (T) 2-2 (T)	*D*_*c*_< ; *D*_*n*_< *D*_*c*_< ; *D*_*n*_>	1 → 19 → No: Allopatric fragmentation

Phylogeographic inferences are inferred by applying the inference key of Posada et al. (2000) (http://darwin.uvigo.es/software/geodis.html)

(T): tip; (I): interior; *D*_*c*_: clade distance; *D*_*n*_: nested clade distance; I-T: interior vs. tip clades

## Discussion

Our analyses using EST-SSR and cpDNA *trnC*-*ycf6* spacer reveal marked population genetic structure and phylogeographic pattern in *Begonia luzhaiensis*, highly concordant with those predicted by Chung et al. (2014). Analyses of EST-SSR (Tables 2 and 3) and cpDNA sequences (Tables 2, 3 and 4) indicated a high level of genetic differentiation and apparent substructure in *B. luzhaiensis* (Fig. 3), suggesting restricted gene flow among isolated populations, as documented in previous studies of *Begonia* (Matolweni et al. 2000; Hughes and Hollingsworth 2008; Nakamura et al. 2014; Twyford et al. 2014; Chan et al. 2018; Li et al. 2018).

### Population diversification in *Begonia luzhaiensis*

Hughes and Hollingsworth (2008) suggest that limited gene flow in *Begonia* could have resulted in several geographic patterns, including geographical restrictions of monophyletic groups, frequent occurrences of narrow endemic species, and relatively few widespread species. Additionally, limited gene flow may greatly enhance effects of random genetic drift, a major mechanism promoting diversification in other large plant genera such as the legume genus *Astragalus* L. (Sanderson and Wojciechowski 1996). Moreover, widespread *Begonia* species show strong population substructure (Matolweni et al. 2000; Hughes and Hollingsworth 2008; Twyford et al. 2013; Chan et al. 2018) and decreased pollen viability in offspring of artificial crossing between isolated populations within species (Twyford et al. 2013, 2014). Here, our observations give further lines of support for this model of population diversification in *Begonia*. *Begonia luzhaiensis*, one of the few widespread species of Clade SVLB, can be grouped into three subpopulations in western, eastern and central Guangxi based on EST-SSR loci (Fig. 3). Given the highly fragmented nature of the cave and cave-like microhabitats (Fig. 1c) across the Sino-Vietnamese limestone karsts inhabited by *B. luzhaiensis*, our results demonstrate that geographic isolation could have played a pivotal role in generating the strong population differentiation of the Clade SVLB as proposed by Chung et al. (2014).

Although the dispersal biology of *Begonia luzhaiensis* has not been investigated, the life history trait and habitat preference of the species fits well with the typical *Begonia*, which is generally a poor seed disperser in sheltered conditions of the forest floor (De Lange and Bouman 1999a, b; Dewitte et al. 2011). Species of *Begonia* sect. *Coelocentrum* inhabit the limestone caves and cave-like microhabitats, which are isolated, closed and have high humidity (Liang et al. 2010). Such habitat preference indicates a great disadvantage for dispersal success in sect. *Coelocentrum*, further limiting effective gene flow.

Given the low dispersal ability and strong niche conservatism of the limestone *Begonia* in the highly fragmented landscape of SVLK, Chung et al. (2014) also predicted restricted gene flow constrained by isolation due to distance in SVLB, a model documented in other low vagility organisms such as amphibians (Kozak et al. 2006; Fouquet et al. 2007). In the current study, Mantel tests of both EST-SSR and cpDNA *trnC*-*ycf6* spacer sequences detect significant positive correlation between genetic and geographic distances (Table 6), congruent with the expectation of isolation by distance in *B. luzhaiensis*. Similarly, NCPA also infers restricted gene flow with isolation by distance as the major mechanism in shaping the phylogeographic structure in populations of western Guangxi (Clade 1-1) (Table 7).

### Causes of phylogeographic patterns

NCPA inferred that phylogeography of *Begonia luzhaiensis* has been shaped predominately by historical fragmentation events, with the exception of Clade 1-1 that “restricted gene flow with isolation by distance” was inferred (Table 7). Liu et al. (2001) distinguishes three karst landforms in Guangxi—karst plain, Fenglin plain, and Fengcong depression (Figs. 2 and 4)—that represent different stages of karstification. Karst plain (I in Figs. 2 and 4) is characterized by scattered residual limestone hills, symbolizing the oldest and last stage of karstification. Fenglin plain (II in Figs. 2 and 4), characterized by isolated, steep-side towers and peaks raising from the plain covered by alluvium and rice paddies, represent the mid stage of Guangxi’s karstification. Fengcong depression (III in Figs. 2 and 4) is the youngest of Guangxi’s karst landform, characterized by conical hills separated by deep, closed depressions. Referring to Liu et al. (2001)’s classification of Guangxi’s karst landforms, haplotypes and clades with inferences of historical fragmentation are predominately distributed in Fenglin plain (II in Fig. 4) where limestone peaks are isolated and largely fragmented, while Clade 1-1 (“restricted gene flow with isolation by distance” inferred by NCPA) is found in Fengcong depression (III in Fig. 4) where depressions separating limestone hills could still function as migration corridors for gene flow. On the other hand, *B. luzhaiensis* is absent from karst plains where limestone hills are highly degraded. Taken together, the phylogeography of *B. luzhaiensis* appears to be correlated well with the karst landform in Guangxi, suggesting the importance of the geohistory of karstification in determining the population genetic structure of *B. luzhaiensis* and thus supporting Chung et al. (2014)’s proposition. While continuous karstification has apparently been crucial in determining the phylogeographic structure of *B. luzhaiensis*, the allopatric fragmentation inferred for the total cladogram is also in line with the mountain chain [i.e., Dananshan, Tianpingshan, Jiaqiaoling, and Dayaoshan (Fig. 4)] that separates Clades 2-1 and 2-2, suggesting that these none-limestone mountains could have created further geographic barriers for gene flow among the fragmented karst landscapes.

### Implications for SVLK plant speciation

Within *B. luzhaiensis*, populations from different caves or cave-like microhabitats have very similar flowers, and yet they display a considerable range of variation in shapes, sizes and variegation in leaves across the species’ distributional range (Figs. 1e–h and 2). This pattern is congruent with other widespread *Begonia* species that all show high levels of morphological differentiation in different populations (Matolweni et al. 2000; Hughes and Hollingsworth 2008; Twyford et al. 2013, 2014; Chan et al. 2018). Dewitte et al. (2011) proposed that the high morphological diversity in *Begonia* could have been explained by adaption to a variety of ecological conditions. However, although shapes and sizes of each cave or cave-like microhabitat seem quite different from one another across the SVLK landscape, major ecological parameters such as temperate, light intensity, humidity, and soil substrate appear to be highly homogeneous (Chung et al. 2014; Monro et al. 2018), characterized by constant temperature, high humidity, and indirect and low light (Liang et al. 2010; Ren et al. 2010; Monro et al. 2018). Additionally, recent studies also show no apparent adaptive difference between *Begonia* species that show different leaf variegation, including several species of Clade SVLB (Zhang et al. 2009; Sheue et al. 2012). Given the marked population genetic structure and phylogeographic pattern, our data also suggest that genetic drift alone could have been sufficient in generating intraspecific leaf variation in *B. luzhaiensis* as shown in other *Begonia* species (Matolweni et al. 2000; Hughes and Hollingsworth 2008; Twyford et al. 2013, 2014). Studies of *Primulina eburnea* (Hance) Yin Z.Wang, a gesnerid species restricted to island-like limestone karsts of South China, has also shown that the marked phylogeographic pattern is mainly driven by genetic drift resulted from fragmented karst landscape (Gao et al. 2015). As the process of karstification in SVLK continues and additional geographic isolation in Clade SVLB is expected, further population differentiation could eventually lead to speciation (Kozak et al. 2006; Fouquet et al. 2007; Gao et al. 2015), resulting in a steady accumulation of species manifesting as non-adaptive species radiation (Gittenberger 1991; Rundell and Price 2009; Chung et al. 2014).

### Anthropogenic effects?

Given that landscape fragmentation triggered by continuous karstification have been crucial in determining the phylogeography and population genetic structure of *Begonia luzhaiensis*, the geohistory of the Sino-Vietnamese limestone karst should have also had a strong influence on the phylogeography of SVLB and other limestone plants. Values of Tajima’s *D* and Fu’s *Fs* did not significant deviate from 0 in the total sample or for each region, suggesting long-term constant population size. Based on results of mismatch distribution and BSP analyses, populations of *B. luzhaiensis* have experienced a stable population size since the species’ expansion from ca. 2.047 Mya (95% confident interval = 0.596–6.072 Mya) until ca. 75,000 years ago (75 Kya) when its effective population size declined, with further population reduction until the present time (Table 5; Fig. 5b).

The prolonged period of stable population size in *B. luzhaiensis* since the late Pliocene to early Pleistocene (Fig. 5b) is congruent with the intensive and accelerated karstification of Sino-Vietnamese limestone karsts ca. 10 to 3 Mya since the late Miocene (Liu 1997; Liu et al. 2001), during which karst landscapes developed, creating abundant caves and cave-like microhabitats for population expansion in *B. luzhaiensis*, most likely from central Guangxi as suggested by the haplotype network (Fig. 4). Not surprisingly, the onset of the last major Northern Hemisphere glacial period ca 20–110 Kya, during which summer temperatures and precipitation reduced drastically due to the weakened summer monsoon (An et al. 2001), jointly retarded the process of karstification in South China (Zhang 1989), likely resulting in a decline of population size in *B. luzhaiensis* 75 Kya (Fig. 5b). However, our data also indicate a continuous and further population declination until present time even though the return of summer monsoons since the end of the last glacial maximum (LGM) should have also resumed the process of karstification, suggesting an alternative cause responsible for the population decline in *B. luzhaiensis* in more recent time.

Paleolithic *Homo sapiens* fossils had been excavated from a large number of sites in China (Wu and Lin 1985; Wu et al. 2012) and Guangxi is one of the most important centers of China’s paleoanthropology, with at least 12 sites reported from Guangxi (Yuan and Wei 2015). Thus far, Guangxi’s Paleolithic human fossils had been excavated from areas including Guilin (Wang et al. 1981), Laibin (Jia and Wu 1959), Lipu (Wu et al. 1962), and Du’an (Zhao et al. 1981) where *B. luzhaiensis* of current study sampled (Fig. 2). All these sites of Guangxi, as well as a majority of paleoanthropological sites in China, are all limestone caves, indicating that caves are not only ideal for the preservation of archaeological remains (Wu et al. 2012; Li et al. 2014; Yuan and Wei 2015) but should have also been important for human activities in the ancient times. However, limestone caves are extremely fragile and highly vulnerable to anthropogenic disturbance (Van Beynen and Townsend 2005; Cao et al. 2015). Our own observations during the field work indicate that *B. luzhaiensis* as well as many other limestone cave plants could only be found in caves with minimal human disturbance (e.g., Fig. 1c).

In South China, the transition from Paleolithic to Neolithic ca. 20–10 Kya marks the change from hunter gathering to food-producing societies (Yuan et al. 1995), with earliest evidence of rice growing in Guangxi dated to ca. 2500–2000 B.C. (Zhang and Hung 2010). Because farmable lands for agriculture are limited in Guangxi’s extensive limestone karsts, the majority of lands between fenglins and fengcongs suitable for rice growing have been transformed into farmlands (Fig. 1b, d). Prior to development, the lands between fenglings and fengcongs should have been heavily forested and contained microhabits that could have functioned as dispersal corridors for SVLK plants such as *B. luzhaiensis*, a conjecture based on observations in residual forests escaping from development. Taken together, the increased anthropogenic activity since the early Holocene ca. 10 Kya, firstly utilizing limestone caves and secondarily transforming dispersal corridor into farmlands, could explain the further population declination in *B. luzhaiensis* until the present time (Table 5; Fig. 5b).

### Conservation of limestone karst and cave flora

Based on EST-SSR and cpDNA sequences, our study suggests both natural and likely anthropogenic causes for the marked population genetic structure in *Begonia luzhaiensis*. To our knowledge, this is the first phylogeographic study providing evidence for the anthropogenic causes for the population declination of a Sino-Vietnamese limestone plant species. Limestone karsts of Southeast Asia and South China have experienced unprecedented destruction, and biodiversity in the region is severely threatened by uncontrolled increases in tourism (Sang et al. 2011), unsustainable limestone quarries (Clements et al. 2006), regional forest loss and degradation (Sodhi et al. 2004; Laurance 2007), and irrational and intensive land use (Wang et al. 2004). Even for species such as *Begonia luzhaiensis* that is not regarded as rare, our followed up survey found that the DL population had disappeared due to cattle grazing. The population in Laibin, where type specimen of *Primulina cardaminifolia* Yan Liu and W.B.Xu was also collected, has vanished due to mud flooding (Xu et al. 2013). Considering these threats to the karst ecosystem, conservation of limestone species is urgently needed (Monro et al. 2018).

Consulting Nekola (1999)’s study, Chung et al. (2014) proposed that cave and cave-like microhabitats are “paleorefugia” of limestone cave flora, representing relics of previously much more widespread microhabitats. Our study reveals a marked genetic structure and declined population size in *B. luzhaiensis*, suggesting that current populations of the species represent remnants of a previously much bigger population, consistent with the idea of paleorefugia. Given this, our study provides important information for developing conservation strategies for limestone species. The genetic isolation and the lack of gene flow among populations of *B. luzhaiensis* suggest that we must pay special attention to small isolated areas with genetic uniqueness because they deserve high conservation priority. Therefore, conservation strategies in karst regions should focus not only on species but also the integrity of their genetic diversity. For example, populations of RS, XP, and GT are distinct genetic groups with no genetic connection with other populations based on cpDNA sequences (Fig. 4), so future management plans should focus on conserving the genetic diversity of these distinct populations. By viewing caves and cave-like microhabitats as paleorefugia, our study urges further investigations into phylogeography and population genetic structures of other SVLK flora to design suitable conservation strategies based on genetic data.

## Conclusions

The microevolution patterns of *B. luzhaiensis* indicate that genetic drift enhanced by limited gene flow could have been the major factor promoting population diversification in *Begonia*, strongly suggesting the importance of extensive karstification in shaping the species diversity of the Sino-Vietnamese limestone karsts. Our phylogeographic analyses also identify the mountain chain composed of Dananshan, Tianpingshan, Jiaqiaoling, and Dayaoshan could further hinder gene flow among fragmented limestone karst habitats. The appearance of the Neolithic human beings and subsequent development of agriculture are shown to be responsible for further destruction of suitable habitats of limestone species, resulting in drastic population declines in more recent time. As most caves and cave-like microhabitats are not under protection, a majority of the SVLK plants are highly threatened by increased and unregulated human activities in the region. We urge prompt and comprehensive studies of SVLK biodiversity because they are essential for the effective conservation of unique and precious flora.

## Additional files

**Additional file 1: Table S1.** Descriptive statistics of EST-SSR loci. **Table S2.** Genetic differentiation (pairwise *F*_ST_) based on EST-SSR between populations of *Begonia luzhaiensis*. All *F*_ST_ values are significant (*P* < 0.001). **Table S3.** Genetic differentiation (pairwise *F*_ST_) *trnC-ycf6* spacer sequence between populations of *Begonia luzhaiensis.* **Table S4.** Pairwise geographical distance [ln (km+1)] between populations of *Begonia luzhaiensis.*

**Additional file 2: Figure S1.** Plot of *F*_ST_ versus heterozygosity (*He*) to identify potential loci subject to selection. The dot in the red zone is the candidate under positive selection, in yellow zone for balancing selection, and in grey zone is neutral. Significance was evaluated at the 5% level.

**Additional file 3: Figure S2.** Demographic history of *Begonia luzhaiensis.* The BSP results from three sampling strategies, including (A) local: east; (B) local: central; (C) local: west; (D) polled; (E) scattered.

## Data Availability

All DNA sequences generated in this study have registered in GenBank.

## Abbreviations

AIC: akaike information criteria
AMOVA: analysis of molecular variance
BSP: Bayesian Skyline Plot
cpDNA: chloroplast DNA
EST-SSR: expressed sequence tag-simple sequence repeat
HWE: Hardy–Weinberg equilibrium
IBD: isolation by distance
LD: linkage disequilibrium
MCMC: Markov chain Monte Carlo
NCPA: nested clade phylogeographic analysis
PCoA: principal coordinate analysis
*SSD*: sum of squared deviations
SVLB: Sino-Vietnamese limestone *Begonia*
SVLK: Sino-Vietnamese limestone karsts
