# Supplementary material for: Phylogeography of Begonia luzhaiensis suggests both natural and anthropogenic causes for the marked population genetic structure
Source: Bot Stud. 2019 Sep 6;60:20. doi: 10.1186/s40529-019-0267-9 (PMC6730737; doi:10.1186/s40529-019-0267-9)
Supplement: Supplementary file 1 — Additional file 1: Table S1. Descriptive statistics of EST-SSR loci. Table S2. Genetic differentiation (pairwise FST) based on EST-SSR between populations of Begonia luzhaiensis. All FST values are significant (P < 0.001). Table S3. Genetic differentiation (pairwise FST) trnC-ycf6 spacer sequence between populations of Begonia luzhaiensis. Table S4. Pairwise geographical distance [ln (km+1)] between populations of Begonia luzhaiensis. [file 40529_2019_267_MOESM1_ESM.docx]

**Supporting information**

**S1 Table.** Descriptive statistics of EST-SSR loci developed in Tseng et al. (2017). All the values are highly significant (*P* < 0.001).

| Locus | *F*_IT_ | *F*_ST_ | *F*_IS_ |
| --- | --- | --- | --- |
| BLZ01 | 0.593 | 0.292 | 0.426 |
| BLZ02 | 0.381 | 0.343 | 0.057 |
| BLZ03 | 0.706 | 0.550 | 0.347 |
| BLZ04 | 0.467 | 0.450 | 0.030 |
| BLZ05 | 0.618 | 0.632 | -0.039 |
| BLZ06 | 0.668 | 0.605 | 0.161 |
| BLZ07 | 0.597 | 0.584 | 0.031 |
| BLZ08 | 0.723 | 0.627 | 0.258 |
| BLZ09 | 0.392 | 0.393 | -0.002 |
| BLZ10 | 0.492 | 0.447 | 0.081 |
| BLZ11 | 0.779 | 0.709 | 0.238 |
| BLZ12 | 0.782 | 0.701 | 0.272 |
| BLZ13 | 0.581 | 0.569 | 0.028 |
| BLZ14 | 0.550 | 0.506 | 0.089 |
| BLZ15 | 0.689 | 0.709 | -0.070 |
| BLZ16 | 0.675 | 0.643 | 0.090 |
| All | 0.589 | 0.527 | 0.131 |

**S2 Table.** Genetic differentiation (pairwise *F*_ST_) based on EST-SSR between populations of *Begonia luzhaiensis*. All *F*_ST_ values are significant (P < 0.001).

| FL | YS | LZ | LG | YF | RS | XC | TE | GT | PL | LY | XP |  |
| --- | --- | --- | --- | --- | --- | --- | --- | --- | --- | --- | --- | --- |
| 0.000 |  |  |  |  |  |  |  |  |  |  |  | FL |
| 0.315 | 0.000 |  |  |  |  |  |  |  |  |  |  | YS |
| 0.378 | 0.428 | 0.000 |  |  |  |  |  |  |  |  |  | LZ |
| 0.376 | 0.365 | 0.427 | 0.000 |  |  |  |  |  |  |  |  | LG |
| 0.513 | 0.431 | 0.479 | 0.443 | 0.000 |  |  |  |  |  |  |  | YF |
| 0.474 | 0.535 | 0.311 | 0.549 | 0.628 | 0.000 |  |  |  |  |  |  | RS |
| 0.560 | 0.489 | 0.435 | 0.579 | 0.585 | 0.551 | 0.000 |  |  |  |  |  | XC |
| 0.489 | 0.483 | 0.432 | 0.596 | 0.630 | 0.608 | 0.434 | 0.000 |  |  |  |  | TE |
| 0.330 | 0.115 | 0.438 | 0.456 | 0.475 | 0.569 | 0.503 | 0.493 | 0.000 |  |  |  | GT |
| 0.509 | 0.530 | 0.434 | 0.655 | 0.676 | 0.637 | 0.537 | 0.345 | 0.557 | 0.000 |  |  | PL |
| 0.608 | 0.520 | 0.567 | 0.644 | 0.635 | 0.659 | 0.503 | 0.508 | 0.513 | 0.604 | 0.000 |  | LY |
| 0.382 | 0.449 | 0.497 | 0.550 | 0.622 | 0.599 | 0.562 | 0.597 | 0.534 | 0.596 | 0.662 | 0.000 | XP |

**S3 Table.** Genetic differentiation (pairwise *F*_ST_) *trnC-ycf6* spacer sequence between populations of *Begonia luzhaiensis.*

| FL | YS | LZ | LG | YF | RS | XC | TE | GT | PL | BM | YZ | FS | DL | LY | XP |  |
| --- | --- | --- | --- | --- | --- | --- | --- | --- | --- | --- | --- | --- | --- | --- | --- | --- |
|  |  |  |  |  |  |  |  |  |  |  |  |  |  |  |  | FL |
| 0.000 |  |  |  |  |  |  |  |  |  |  |  |  |  |  |  | YS |
| 0.005 | 0.005 |  |  |  |  |  |  |  |  |  |  |  |  |  |  | LZ |
| 0.004 | 0.004 | 0.004 |  |  |  |  |  |  |  |  |  |  |  |  |  | LG |
| 0.002 | 0.002 | 0.002 | 0.002 |  |  |  |  |  |  |  |  |  |  |  |  | YF |
| 0.007 | 0.007 | 0.003 | 0.007 | 0.005 |  |  |  |  |  |  |  |  |  |  |  | RS |
| 0.005 | 0.005 | 0.000 | 0.004 | 0.002 | 0.003 |  |  |  |  |  |  |  |  |  |  | XC |
| 0.005 | 0.005 | 0.000 | 0.004 | 0.002 | 0.003 | 0.000 |  |  |  |  |  |  |  |  |  | TE |
| 0.002 | 0.002 | 0.002 | 0.002 | 0.000 | 0.005 | 0.002 | 0.002 |  |  |  |  |  |  |  |  | GT |
| 0.007 | 0.007 | 0.002 | 0.007 | 0.005 | 0.005 | 0.002 | 0.002 | 0.005 |  |  |  |  |  |  |  | PL |
| 0.005 | 0.005 | 0.000 | 0.004 | 0.002 | 0.003 | 0.000 | 0.000 | 0.002 | 0.002 |  |  |  |  |  |  | BM |
| 0.005 | 0.005 | 0.000 | 0.004 | 0.002 | 0.003 | 0.000 | 0.000 | 0.002 | 0.002 | 0.000 |  |  |  |  |  | FS |
| 0.007 | 0.007 | 0.002 | 0.007 | 0.005 | 0.005 | 0.002 | 0.002 | 0.005 | 0.000 | 0.002 | 0.002 |  |  |  |  | DL |
| 0.006 | 0.006 | 0.001 | 0.006 | 0.003 | 0.004 | 0.001 | 0.001 | 0.003 | 0.001 | 0.001 | 0.001 | 0.001 |  |  |  | YZ |
| 0.005 | 0.005 | 0.000 | 0.004 | 0.002 | 0.003 | 0.000 | 0.000 | 0.002 | 0.002 | 0.000 | 0.000 | 0.002 | 0.001 |  |  | LY |
| 0.002 | 0.002 | 0.006 | 0.006 | 0.004 | 0.009 | 0.006 | 0.006 | 0.004 | 0.009 | 0.006 | 0.006 | 0.009 | 0.007 | 0.006 |  | XP |

**S4 Table.** Pairwise geographical distance [ln (km+1)] between populations of *Begonia luzhaiensis.*

| FL | YS | LZ | LG | YF | RS | XC | TE | GT | PL | BM | FS | DL | YZ | LY | XP |  |
| --- | --- | --- | --- | --- | --- | --- | --- | --- | --- | --- | --- | --- | --- | --- | --- | --- |
| 0.000 |  |  |  |  |  |  |  |  |  |  |  |  |  |  |  | FL |
| 2.010 | 0.000 |  |  |  |  |  |  |  |  |  |  |  |  |  |  | YS |
| 4.479 | 4.407 | 0.000 |  |  |  |  |  |  |  |  |  |  |  |  |  | LZ |
| 3.758 | 3.695 | 4.196 | 0.000 |  |  |  |  |  |  |  |  |  |  |  |  | LG |
| 4.991 | 4.952 | 4.181 | 4.737 | 0.000 |  |  |  |  |  |  |  |  |  |  |  | YF |
| 4.930 | 4.891 | 4.133 | 4.635 | 2.817 | 0.000 |  |  |  |  |  |  |  |  |  |  | RS |
| 5.325 | 5.293 | 4.837 | 5.254 | 4.727 | 4.849 | 0.000 |  |  |  |  |  |  |  |  |  | XC |
| 5.842 | 5.825 | 5.553 | 5.748 | 5.300 | 5.359 | 5.202 | 0.000 |  |  |  |  |  |  |  |  | TE |
| 2.542 | 2.174 | 4.406 | 3.839 | 4.964 | 4.908 | 5.278 | 5.826 | 0.000 |  |  |  |  |  |  |  | GT |
| 5.856 | 5.838 | 5.569 | 5.777 | 5.355 | 5.419 | 5.110 | 3.890 | 5.836 | 0.000 |  |  |  |  |  |  | PL |
| 5.788 | 5.768 | 5.477 | 5.700 | 5.235 | 5.307 | 4.989 | 3.804 | 5.767 | 3.219 | 0.000 |  |  |  |  |  | BM |
| 5.820 | 5.800 | 5.522 | 5.745 | 5.319 | 5.388 | 4.981 | 4.269 | 5.797 | 3.320 | 3.455 | 0.000 |  |  |  |  | FS |
| 5.866 | 5.848 | 5.583 | 5.788 | 5.373 | 5.436 | 5.123 | 3.960 | 5.846 | 1.708 | 3.372 | 3.305 | 0.000 |  |  |  | DL |
| 5.304 | 5.272 | 4.741 | 5.180 | 4.351 | 4.536 | 3.911 | 5.033 | 5.266 | 5.011 | 4.849 | 4.918 | 5.033 | 0.000 |  |  | YZ |
| 5.955 | 5.938 | 5.698 | 5.881 | 5.507 | 5.561 | 5.305 | 4.120 | 5.937 | 3.624 | 4.099 | 4.065 | 3.548 | 5.228 | 0.000 |  | LY |
| 2.675 | 2.839 | 4.480 | 3.516 | 4.967 | 4.895 | 5.343 | 5.836 | 3.208 | 5.855 | 5.786 | 5.821 | 5.865 | 5.307 | 5.953 | 0.000 | XP |
