# Supplementary figures and images for: Phylogeography of Begonia luzhaiensis suggests both natural and anthropogenic causes for the marked population genetic structure
Source: Bot Stud. 2019 Sep 6;60:20. doi: 10.1186/s40529-019-0267-9 (PMC6730737; doi:10.1186/s40529-019-0267-9)

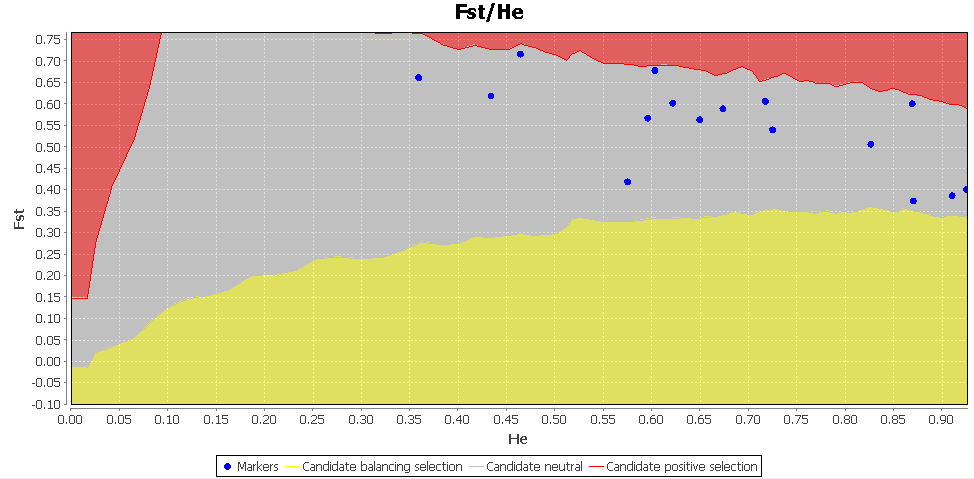

Supplement: Supplementary file 2 — Additional file 2: Figure S1. Plot of FST versus heterozygosity (He) to identify potential loci subject to selection. The dot in the red zone is the candidate under positive selection, in yellow zone for balancing selection, and in grey zone is neutral. Significance was evaluated at the 5% level. [file 40529_2019_267_MOESM2_ESM.tif]

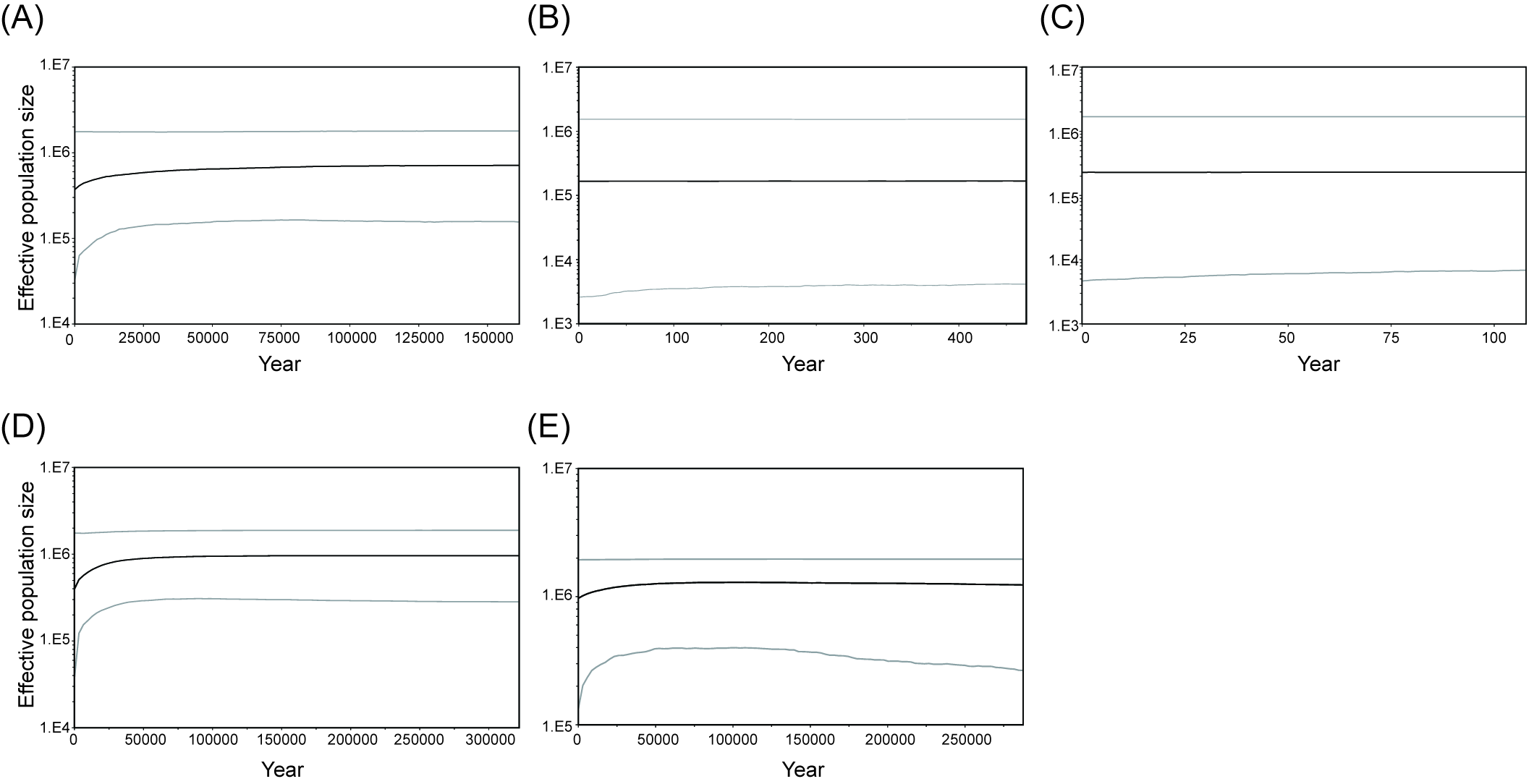

Supplement: Supplementary file 3 — Additional file 3: Figure S2. Demographic history of Begonia luzhaiensis. The BSP results from three sampling strategies, including (A) local: east; (B) local: central; (C) local: west; (D) polled; (E) scattered. [file 40529_2019_267_MOESM3_ESM.tif]
